# Effectiveness of exercise in reducing symptom burden among hemodialysis patients: a non-pharmacological intervention approach

**DOI:** 10.3389/fpubh.2025.1580689

**Published:** 2025-07-09

**Authors:** Bushra Alshammari, J. Silvia Edison, Sameer A. Alkubati, Awatif M. Alrasheeday, Bander Albagawi, Laila Lafi Alharbi, Hajer I. Motakef, Layla Alshammari, Bahia Galal Abd El-Razik Siam, Nawal Abdullah Alharbi, Wejdan Assiri, Amirah Abdulaziz Almoqad, Abdulrahman Ibrahim Aldibas, Farhan Alshammari

**Affiliations:** ^1^Medical Surgical Nursing Department, College of Nursing, University of Hail, Hail, Saudi Arabia; ^2^Nursing Administration Department, College of Nursing, University of Hail, Hail, Saudi Arabia; ^3^Medical Surgical Department, College of Nursing, Imam Mohammad Ibn Saud Islamic University, Riyadh, Saudi Arabia; ^4^King Salman Specialist Hospital, Emergency Department Director, Hail Health Cluster, Hail, Saudi Arabia; ^5^Maternal and Child Health Department, College of Nursing, University of Hail, Hail, Saudi Arabia; ^6^Academic Affairs and Medical Education, Maternity and Children’s Hospital, Hail Health Cluster, Ministry of Health, Hail, Saudi Arabia; ^7^Critical Care Nursing Program, Ministry of Health, Riyadh, Saudi Arabia; ^8^Quality Department, Riyadh Health Cluster, Riyadh, Saudi Arabia; ^9^King Saud Medical City, Riyadh, Saudi Arabia; ^10^Department of Pharmaceutics, College of Pharmacy, University of Hail, Hail, Saudi Arabia

**Keywords:** end-stage renal disease, hemodialysis, exercise intervention, symptom burden, non-pharmacological intervention, quality of life

## Abstract

**Background:**

End-stage renal disease (ESRD) patients undergoing hemodialysis (HD) often experience a substantial symptom burden, which negatively impacts their quality of life. While pharmacological treatments are commonly used to manage these symptoms, non-pharmacological interventions, such as exercise, have gained attention for their potential to alleviate both physical and psychological symptoms without additional medication-related side effects. Compared to other strategies that often target a limited range of symptoms—such as meditation for depression or music therapy for anxiety—exercise offers broader benefits, making it particularly promising for patients receiving HD.

**Objective:**

This study aimed to evaluate the effectiveness of an exercise intervention in reducing the symptom burden among patients receiving HD in Hail City, Saudi Arabia.

**Methods:**

A quasi-experimental pre-post intervention design was employed, involving (n = 72) HD patients recruited through convenience sampling from King Salman Specialist Hospital and King Khalid Hospital. Participants engaged in a structured exercise program for 12 weeks, with sessions conducted three times per week for 30 min. Symptom severity was assessed using the Dialysis Symptom Index (DSI) before and after the intervention. Data were analyzed using paired t-tests, with a significance level set at *p* < 0.05.

**Results:**

A total of 72 HD patients participated in the study. The exercise intervention led to a statistically significant reduction in overall symptom severity scores, decreasing from 105.94 ± 9.77 pre-intervention to 102.50 ± 9.61 post-intervention (*p* < 0.001). Significant improvements were noted in multiple symptoms, including constipation (*p* = 0.002), decreased appetite (p < 0.001), restless legs (*p* = 0.001), fatigue (*p* < 0.001), joint pain (*p* = 0.045), difficulty concentrating (*p* = 0.045), and several emotional symptoms such as worrying (*p* = 0.024), nervousness (*p* = 0.002), trouble sleeping (*p* < 0.05), and feelings of sadness (*p* < 0.001). Sociodemographic factors, including younger age, female gender, shorter dialysis duration, and higher comorbidity burden, were significantly associated with greater baseline symptom severity.

**Conclusion:**

The findings demonstrate that a structured exercise program is an effective non-pharmacological intervention for reducing symptom burden among HD patients. These results support incorporating exercise into routine HD care to enhance patient well-being. Future initiatives should focus on integrating supervised, accessible exercise programs into dialysis centers to maximize adherence and therapeutic benefit.

## Introduction

End-stage kidney disease (ESRD) is a critical condition characterized by the irreversible loss of kidney function, requiring renal replacement therapy to sustain life. Globally, the estimated number of individuals with ESKD requiring renal replacement therapy ranges from 4.902 million to 9.701 million (1). The prevalence of ESKD has been on the rise due to the increasing incidence of diabetes mellitus, hypertension, and other chronic illnesses, which pose a significant public health challenge (2). The management of ESKD primarily involves two treatment modalities: kidney transplantation and dialysis. Kidney transplantation is widely regarded as the optimal therapeutic option due to its ability to restore normal renal function and enhance long-term patient outcomes (3, 4). However, it is often limited by a shortage of donor organs, strict eligibility criteria, healthcare budget constraints, and the risk of complications such as rejection and infection. As a result, the majority of patients with ESKD rely on dialysis as a life-sustaining treatment (4–6).

Among the dialysis modalities, hemodialysis (HD) is the most prevalent, accounting for approximately 90% of patients undergoing renal replacement therapy (7). HD is a life-saving intervention that removes toxins, excess fluids, and waste products from the blood, mimicking the filtering function of healthy kidneys (8). While HD is essential for survival, it is associated with a wide range of physical, psychological, and emotional challenges due to the symptoms they experience. Symptom burden is, therefore, ‘the sum of the severity and impact of symptoms reported by a significant proportion of patients with a given disease or treatment’ (9). According to comprehensive reviews, the average number of symptoms per patient ranges between 6 and 20, including fatigue, muscle cramps, sleep disturbances, gastrointestinal discomfort, dry skin, and itching (10). Furthermore, HD patients frequently report psychological distress, including feelings of sadness (11), anxiety, irritability, and reduced quality of life (12–14). These symptoms not only affect daily functioning but also contribute to increased morbidity and healthcare utilization (15).

Given the substantial symptom burden associated with HD, there is a growing need for effective management strategies that extend beyond pharmacological treatments (16, 17). While medications play a key role in managing specific symptoms, non-pharmacological interventions are gaining attention due to their potential to address multiple symptoms holistically without the risk of drug interactions or side effects.

The need for effective treatments to address hemodialysis-related symptoms has been widely acknowledged as a critical knowledge gap and a key research priority by patients, caregivers, and healthcare professionals (4). Exercise is a practical and promising approach to reducing symptom burden, with existing literature supporting its benefits. Studies have shown a clear association between low physical activity and a higher prevalence of symptoms among individuals on dialysis (18, 19). Notably, even in patients with limited physical function, structured exercise programs are safe and have been shown to improve outcomes closely linked to symptom burden, including functional status and health-related quality of life (20, 21). Regular physical exercise, in particular, has been increasingly recognized as a valuable intervention for alleviating the symptom burden among patients with chronic illnesses, including those receiving HD (22). Exercise has demonstrated significant benefits in improving physical functioning, enhancing cardiovascular health, increases muscle strength, reduces inflammation, and mitigates symptoms such as fatigue, muscle cramps, and joint stiffness commonly experienced by HD patients (23). According to recent reviews and meta-analyses, exercise has been shown to effectively reduce anxiety, depressive symptoms and emotional distress, highlighting its potential as a valuable non-pharmacological approach to improve psychological well-being (22, 23). By addressing both physical and psychological dimensions of health, exercise targets a broader spectrum of symptoms experienced by HD patients than most other non-pharmacological strategies, making it a particularly effective and comprehensive approach to enhancing overall patient well-being.

Exercise may reduce symptom burden in HD patients through several mechanisms. Improved circulation and muscle oxygenation can alleviate fatigue and restless legs, while enhanced flexibility and strength may ease joint pain. Additionally, exercise stimulates endorphin release and modulates neurotransmitters, contributing to reduced anxiety, depression, and sleep disturbances (22, 24).

The exercise program adopted in this study—three sessions per week over a 12-week period—was informed by previous research demonstrating that such frequency and duration are both feasible and effective in eliciting meaningful improvements in physical function and psychological well-being among HD patients (20, 23). Meta-analyses have further shown that interventions lasting longer than 8 weeks are sufficient to achieve significant reductions in fatigue, depressive symptoms, and musculoskeletal discomfort in this population (21).

In the context of Hail City, Saudi Arabia, several cultural and environmental factors may influence the feasibility of exercise among HD patients. Social norms, particularly among women, may limit participation in outdoor or public exercise settings due to cultural modesty standards. Moreover, extreme summer temperatures and limited access to climate-controlled public fitness facilities can restrict outdoor physical activity (25). Despite these challenges, the growing national emphasis on healthy lifestyles under Saudi Vision 2030 has increased public awareness of the benefits of physical activity, even among chronically ill populations (26). These regional dynamics underscore the need for culturally sensitive and adaptable exercise interventions that can be implemented within clinical settings or at home.

This study aims to evaluate the effectiveness of exercise in reducing the symptom burden among patients undergoing HD. By examining the relationship between exercise and symptom severity, and identifying demographic and clinical factors influencing this relationship, the findings aim to provide evidence-based insights for incorporating exercise as a routine non-pharmacological strategy to improve patient outcomes and quality of life. It is hypothesized that participation in a structured 12-week exercise program will result in a significant reduction in symptom burden among patients undergoing HD.

## Method

### Study design

This study employed a quasi-experimental pre-post intervention design to assess the effectiveness of exercise in reducing symptom burden among HD patients. The study compared symptom severity before and after the intervention to evaluate the impact of exercise on physical and psychological symptoms in this population. The study was conducted over a six-month period, from May 2024 to November 2024.

### Setting

The study was conducted at King Salman Specialist Hospital and King Khalid Hospital in Hail City, Saudi Arabia. These government-operated medical institutions provide free healthcare services and emergency care exclusively to Saudi nationals. Both hospitals have a high patient capacity and offer treatment across multiple specialties, including HD services as part of their specialized medical care. The HD centers within these hospitals provide life-sustaining dialysis treatment for patients with ESKD and collectively accommodate approximately 500 patients per week. Operating 6 days a week, the centers organize dialysis sessions into two daily shifts—morning and evening—to optimize service delivery and patient access.

### Sample and sample size calculation

A convenience sampling method was used to recruit patients undergoing HD at King Salman Specialist Hospital and King Khalid Hospital in Hail City, Saudi Arabia. To minimize selection bias, a standardized screening process was applied to all potential participants, and an independent renal unit staff member facilitated recruitment to ensure consistent application of inclusion and exclusion criteria across both sites.

The required sample size for this study was determined using G*Power software for a paired t-test (pre-post intervention design) to assess the effectiveness of exercise in reducing symptom burden among HD patients. Based on previous studies evaluating the impact of exercise interventions on symptom burden in HD patients, an expected effect size (Cohen’s d) of 0.5 was selected, representing a moderate effect (27). To achieve a statistical power of 80% (*β* = 0.20) and a significance level of 0.05 (*α* = 0.05), a paired t-test sample size calculation indicated a minimum of 33 participants. Given the potential for patient dropouts, non-compliance, or loss to follow-up, an additional 20% was added to the required sample size, bringing the final recommended sample size to approximately 40 participants.

Patients were eligible for inclusion if they were 18 years or older, had been undergoing maintenance HD for at least 6 months, and were medically cleared to engage in light-to-moderate exercise as assessed by their nephrologist. Participants were required to provide written informed consent before enrollment.

Patients with severe cardiovascular disease, including uncontrolled hypertension or a recent history of cardiac events, were excluded due to the potential risks associated with physical activity. Additionally, individuals with musculoskeletal or neurological conditions that impaired mobility, as well as those with cognitive impairment or psychiatric disorders that could hinder adherence to the intervention, were not included. Patients who were unable to comply with the exercise program or who withdrew from the study at any stage were also excluded.

### Recruitment process and exercise intervention

The recruitment process was facilitated by a designated renal unit staff member, who acted as a gatekeeper in pre-screening and identifying eligible patients at the HD centers. Patients who met the inclusion and exclusion criteria were informed about the study, and if they expressed interest, the gatekeeper introduced them to the researcher. Each potential participant received an information sheet detailing the study objectives, procedures, and potential benefits, along with a consent form. Patients willing to participate were required to sign and return the consent form, granting permission to be included in the study. Upon obtaining informed consent, the researcher provided further explanations and assistance, ensuring that participants fully understood the study process. Following this, patients completed a pre-intervention questionnaire to assess their baseline symptom burden.

Participants were provided with an exercise guide and instructed to perform exercises three times per week for a minimum of 30 min per session. To accommodate varying levels of physical endurance, they were advised that if completing 30 consecutive minutes was challenging, they could break the session into three 10-min intervals throughout the day.

Exercise sessions were conducted in two different settings based on participant preference. Some patients opted to perform their exercises during dialysis sessions, utilizing the time spent receiving treatment to engage in structured physical activity. Others preferred to exercise on non-dialysis days, particularly the day after their sessions, as they reported experiencing dizziness immediately post-dialysis.

For participants who chose to exercise at home, adherence was monitored by the researcher through weekly phone calls to their personal mobile numbers. During these calls, patients were asked to report the frequency, duration, and any challenges faced during their exercise routines. Additionally, they were encouraged to seek support if they experienced any discomfort or difficulty maintaining consistency.

Although exercise sessions were unsupervised, patients were instructed to self-monitor for signs of excessive fatigue or pain using a simple 0–10 rating scale and were advised to discontinue activity and contact the research team if their discomfort exceeded moderate levels (i.e., a score >5). Weekly follow-up calls also included questions about pain and post-exercise fatigue to detect potential adverse responses.

The exercise regimen followed a structured approach, incorporating aerobic exercises, resistance training, muscle-strengthening exercises, and flexibility exercises, as outlined in the provided exercise guide. Each session consisted of a warm-up (5–10 min), a main exercise routine (15–20 min), and a cool-down (5–10 min) to ensure safety and gradual progression. This structured yet flexible approach enabled participants to integrate exercise into their daily routine while accommodating their physical abilities and individual preferences.

### Questionnaire

Demographic and health-related data were collected, including age, gender, marital status, educational level, and employment status. Clinical variables such as dialysis duration and comorbidities were obtained from dialysis charts or hospital records. Comorbidity was assessed using the Davies Comorbidity Index (DCI) to ensure standardized evaluation (28).

The DCI assigns one point per condition, with a maximum possible score of 7 (28). Based on the total score, patients are categorized into three risk groups: Grade 0 (score = 0), Grade 1 (score = 1–2), and Grade 2 (score = 3–7). This index was selected for the study due to its simplicity in structure and scoring, making it a practical tool for assessing comorbidity burden in clinical settings.

The 30-item Dialysis Symptom Index (DSI) was used to assess the presence and severity of physical and emotional symptoms among patients undergoing HD before and after the intervention. The DSI, developed by Weisbord et al. (29), is a widely utilized instrument for evaluating symptom burden in dialysis patients (10). Although originally designed for individuals undergoing dialysis, it has also been successfully applied in studies involving patients with other chronic illnesses, such as cancer, to assess symptom prevalence and severity (29).

Patients were required to indicate which of the 30 listed symptoms they had experienced within the past 7 days. For each symptom reported as present, they rated its severity using a five-point Likert scale, ranging from “not at all bothersome” to “very bothersome.”

To quantify the overall symptom burden, a symptom burden score was calculated by summing the total number of symptoms reported, yielding a possible score range of 0 to 30. Additionally, a symptom severity score was derived by summing the severity ratings of all symptoms, with symptoms not present assigned a score of zero, resulting in a total possible score range of 0 to 150. The reliability and validity of the DSI, including its test–retest reliability, content validity, and construct validity, have been established in previous studies involving patients on HD (29, 30).

### Data analysis

Statistical analysis was conducted using SPSS version 27 to compare pre- and post-intervention symptom severity among HD patients. Descriptive statistics, including means, standard deviations, frequencies, and percentages, were used to summarize patient characteristics and symptom severity scores before and after the exercise intervention.

A paired t-test was performed to assess differences in symptom severity scores before and after the 12-week exercise intervention, determining whether the changes were statistically significant. Additionally, independent t-tests and one-way ANOVA were employed to examine the relationships between symptom severity and sociodemographic and clinical variables, such as age, gender, dialysis access type, and comorbidities. A *p*-value of <0.05 was considered statistically significant.

### Ethical consideration

This study was conducted in accordance with ethical guidelines and received ethical approval from the Institutional Review Board (IRB) of the University of Hail (Approval No H-2024-203). Prior to participation, all eligible patients were provided with detailed information about the study objectives, procedures, potential benefits, and any associated risks. Written informed consent was obtained from all participants, ensuring their voluntary participation and right to withdraw from the study at any stage without consequences.

To ensure confidentiality and data protection, all patient information was anonymized by assigning unique identification numbers, preventing the disclosure of any personal identifiers. Access to the coded data was restricted to a limited number of researchers solely for the purpose of linking pre- and post-intervention results. This measure ensured compliance with ethical research standards and safeguarded participant privacy throughout the study.

Any participant experiencing discomfort or adverse effects during the intervention was advised to discontinue the exercise immediately, and an urgent medical referral was arranged as necessary. Additionally, participants were informed that the exercise intervention was designed to be safe and tailored to their physical condition, with continuous monitoring by highly qualified specialized staff to minimize potential risks and ensure their well-being throughout the study.

## Results

A total of 72 patients participated in the study. Most participants were over the age of 50 (72.2%, *n* = 52), while 27.8% (*n* = 20) were younger than 50 years. The gender distribution showed that 55.6% (*n* = 40) were male and 44.4% (*n* = 32) were female. Regarding educational background, 51.4% (*n* = 37) had completed secondary education, 25.0% (*n* = 18) had a university degree, and 23.6% (*n* = 17) had only elementary education. In terms of dialysis duration, the majority (63.9%, *n* = 46) had been on dialysis for 1–5 years, followed by 29.2% (*n* = 21) for 6–10 years, and 6.9% (*n* = 5) for more than 10 years. As for employment status, 80.6% (*n* = 58) of participants were unemployed, while 19.4% (*n* = 14) were employed.

As shown in Table 1, there was a significant relationship between symptom severity and age, gender, dialysis duration, and comorbidity (*p* = 0.035, 0.020, 0.002, and 0.040, respectively). Younger patients (<50 years) reported a higher symptom burden (109.85 ± 7.30) compared to older patients (104.44 ± 10.24, *p* = 0.035). Females experienced more severe symptoms (108.90 ± 8.23) than males (103.57 ± 10.35, *p* = 0.020). Similarly, patients with shorter dialysis duration (1–5 years) had the highest symptom burden (108.23 ± 10.63), which significantly decreased as dialysis duration increased (*p* = 0.002).

**Table 1:** Relationship between patients’ sociodemographic characteristics and the severity of symptoms.

Variables	Categories	*N* = (72)	%	Mean ± SD	*t/f*	df	(*p*-value)
Age	54.00 ± 9.40						
	<50	20	27.8	109.85 ± 7.30	2.15	70	0.035
	More than 50	52	72.2	104.44 ± 10.24			
Gender	Male	40	55.6	103.57 ± 10.35	−2.37	70	0.020
	Female	32	44.4	108.90 ± 8.23			
Educational level	Elementary	17	23.6	101.58 ± 10.96	2.47	69	0.092
	Secondary	37	51.4	106.75 ± 9.01			
	University	18	25.0	108.38 ± 9.35			
Dialysis duration/years	1–5	46	63.9	108.23 ± 10.63	69	6.63	0.002
	6–10	21	29.2	103.85 ± 4.80			
	>10	5	6.9	93.60 ± 6.02			
Employment	Yes	14	19.4	109.28 ± 9.65	70	1.43	0.156
	No	58	80.6	105.13 ± 9.71			
Comorbidity	0	19	26.4	100.21 ± 7.25	68	2.91	0.040
	1–2	20	27.8	103.41 ± 7.24			0.040
	3–7	33	45.8	105.06 ± 11.63			

t, independent sample *t*-test; f, ANOVA test.

Regarding comorbidities, patients with multiple diseases (3–7 comorbidities) had the highest symptom burden (105.06 ± 11.63), followed by those with 1–2 comorbidities (103.41 ± 7.24) and those with no comorbidities (100.21 ± 7.25, *p* = 0.040). However, educational level and employment status were not significantly associated with symptom severity (*p* > 0.05). No adverse events were reported by any participants during or following the exercise sessions over the 12-week intervention period.

Table 2 illustrates the severity of symptoms pre and post-intervention. There was a significant decrease in the severity of symptoms post-intervention for the following symptoms; constipation, decreased appetite, restless legs, feeling tired-fatigue, joint pain, difficulty of concentration, worrying, feeling nervous, trouble falling asleep, trouble staying asleep, feeling irritable, feeling sad, and feeling anxious (*p* < 0.05). The comparison of pre- and post-exercise intervention scores revealed a statistically significant reduction in overall severity scores. The mean score decreased from 105.94 ± 9.77 before the intervention to 102.50 ± 9.61 after the exercise intervention, with a *p*-value of < 0.001, indicating a significant improvement following the intervention.

**Table 2:** Patients’ symptoms severity pre and post-intervention (*N* = 72).

Symptoms	Pre-intervention	Post-intervention	t	df	*p*-value
	Mean ± SD	Mean ± SD			
Constipation	2.00 ± 1.48	1.84 ± 1.32	3.247	71	0.002
Nausea	3.41 ± 1.92	3.37 ± 0.22	1.757	71	0.078
Vomiting	3.22 ± 2.05	2.59 ± 1.81	2.044	71	0.083
Diarrhea	3.33 ± 2.05	3.31 ± 2.04	1.000	71	0.321
Decrease appetite	3.50 ± 1.81	2.30 ± 1.70	9.591	71	<0.001
Muscle cramps	3.52 ± 1.92	3.48 ± 1.90	1.757	71	0.081
Swelling in legs	3.73 ± 1.65	3.69 ± 1.63	1.757	71	0.083
Shortness of breath	3.50 ± 1.85	3.47 ± 1.83	1.424	71	0.159
Lightheadedness	4.08 ± 1.67	4.04 ± 1.66	1.757	71	0.085
Restless legs	4.22 ± 0.98	4.08 ± 0.96	3.384	71	0.001
Numbness	3.65 ± 1.72	3.62 ± 1.71	1.424	71	0.149
Feeling tired—Fatigue	4.13 ± 1.59	3.87 ± 1.59	3.567	71	<0.001
Cough	3.34 ± 2.00	3.30 ± 1.94	1.757	71	0.083
Dry mouth	3.43 ± 2.06	3.38 ± 1.98	1.757	71	0.079
Bone or joint pain	3.44 ± 1.66	2.38 ± 1.64	2.044	71	0.045
Chest pain	1.62 ± 1.91	1.59 ± 1.87	1.424	71	0.159
Headache	3.86 ± 1.41	3.81 ± 1.40	1.757	71	0.083
Muscle soreness	3.61 ± 1.87	3.58 ± 1.86	1.424	71	0.159
Difficulty of concentration	3.38 ± 1.93	3.33 ± 1.90	2.044	71	0.045
Dry skin	3.93 ± 1.76	3.88 ± 1.76	1.757	71	0.086
Itching	2.84 ± 1.88	2.81 ± 1.87	1.424	71	0.159
Worrying	3.95 ± 1.42	3.88 ± 1.44	2.302	71	0.024
Feeling nervous	3.70 ± 1.80	3.51 ± 1.76	3.166	71	0.002
Trouble falling asleep	3.37 ± 2.28	3.15 ± 2.20	3.090	71	0.003
Trouble staying asleep	3.63 ± 1.60	3.52 ± 1.57	2.044	71	0.045
Feeling irritable	3.86 ± 1.62	3.79 ± 1.61	1.688	71	0.096
Feeling sad	3.69 ± 1.85	3.33 ± 1.75	3.955	71	<0.001
feeling anxious	3.61 ± 1.64	3.55 ± 1.63	1.653	71	0.103
Decrease interest of sex	3.90 ± 1.58	3.87 ± 1.60	1.424	71	0.159
Difficulty becoming Sexually aroused	4.48 ± 0.83	4.44 ± 0.85	1.757	71	0.083
Total	105.94 ± 9.77	102.50 ± 9.61	12.539	71	<0.001

Paired t-test; SD, Standard deviation.

## Discussion

This study examined the relationship between patients’ sociodemographic and clinical characteristics and the severity of symptoms among individuals undergoing HD. Additionally, it assessed the impact of an exercise intervention on symptom severity. The findings revealed significant associations between age, gender, dialysis duration, and comorbidities with symptom burden. Furthermore, the intervention resulted in a significant reduction multiple physical and emotional symptoms among patients receiving HD.

### Relationship between sociodemographic factors and symptom burden

The results indicated that younger patients, females, and those with longer dialysis duration reported higher symptom severity compared to their counterparts. While the association between younger age and greater symptom burden may seem counterintuitive, it has been well-documented in previous research. Younger patients often experience greater psychological distress due to dialysis-related lifestyle restrictions, which may heighten their perception of symptom severity (31, 32). The challenges younger individuals face in adapting to dialysis-related restrictions, along with social and financial disruptions compared to their peers, may further contribute to this burden. Additionally, Jeong Lee and Jeon (32) highlighted that younger patients exhibit higher emotional instability, energy insufficiency, and weakness, exacerbating their symptom burden. Furthermore, age was inversely associated with anxiety and depression, suggesting that younger patients experience greater psychological distress (33).

However, these findings contrast with the majority of studies that found age to be significantly associated with symptom burden, with older patients reporting a higher symptom load (34, 35). This discrepancy may be related to age-related health deterioration and the higher prevalence of chronic illnesses such as diabetes and hypertension, both of which are commonly associated with increased symptom burden (36). Similarly, female patients exhibited higher symptom severity, consistent with previous research indicating that women report a greater symptom burden (34, 37). This disparity may be attributed to differences in pain perception, hormonal influences, and psychological factors (38). Additionally, evidence suggested that women are more likely to express discomfort and seek medical attention, in contrast to the more reserved approach observed in male patients (39, 40). Furthermore, it has been proposed that higher stress and depression levels in women contribute to the increased symptom burden observed in this group (41).

This study found that higher symptom burden levels were associated with higher comorbidity scores, which aligns with previous research (33, 39, 42). Patients undergoing dialysis often experience multiple symptoms simultaneously, stemming from various sources, including renal failure (e.g., restless legs, pruritus), dialysis treatment (e.g., cramping, intradialytic hypotension, sleep disturbances), and comorbid conditions. For example, diabetes is commonly associated with diabetic peripheral neuropathy, leading to numbness, tingling, and pain in the extremities (43). The presence of multiple comorbidities has been identified as a key factor influencing symptom burden in patients with CKD (44), underscoring the need for integrated management approaches to address both primary renal symptoms and those arising from coexisting conditions.

The findings of this study indicate a significant relationship between dialysis duration and symptom burden, with patients undergoing dialysis for 1–5 years experiencing the highest symptom distress, which gradually decreased as dialysis duration increased. However, these results contrast with the findings of Yu et al. (44), who reported a positive association between longer dialysis duration and higher symptom distress. Some research indicates that patients with shorter dialysis durations experience higher symptom distress, which decreases over time, possibly due to physiological adaptation or improved symptom management. For instance, a study found that symptom burden worsened considerably before dialysis initiation and stabilized afterward (45). Conversely, other studies have reported a positive association between longer dialysis duration and increased symptom burden, suggesting that prolonged exposure to dialysis may lead to cumulative physical and psychological stressors (39). These findings highlight the importance of individualized symptom management strategies, considering both early-stage and long-term dialysis patients, to optimize their quality of life and treatment outcomes.

### Impact of the exercise intervention on symptom severity

Following the exercise intervention, a statistically significant reduction in overall symptom severity was observed, with an average decrease of approximately 3.4 points across multiple symptoms. These included constipation, decreased appetite, restless legs, fatigue, joint pain, difficulty concentrating, sleep disturbances, and emotional symptoms such as worrying, nervousness, irritability, sadness, and anxiety. These findings align with prior research demonstrating the beneficial effects of exercise on both physical and psychological well-being in HD patients (46). Although the reduction in the total symptom burden score was modest (approximately 3.4 points), it may still hold clinical significance. In patients undergoing chronic HD, even small improvements in symptom severity—particularly in fatigue, pain, or emotional distress—can enhance daily functioning, treatment adherence, and perceived quality of life. Previous literature has indicated that symptom reductions of this magnitude may be meaningful when distributed across multiple symptom domains, especially in complex chronic populations (21, 30). Thus, the findings suggest a potentially valuable role for structured exercise in multidimensional symptom relief.

Among the physical symptoms, fatigue and joint pain showed significant improvement post-intervention. Fatigue is a well-documented concern among HD patients, often linked to chronic inflammation, reduced physical activity, and anemia (47). The observed reduction in fatigue suggests that exercise may play a role in enhancing circulatory efficiency and muscle function, thereby reducing feelings of tiredness. Similarly, the improvement in joint pain may be attributed to the role of physical activity in enhancing mobility, reducing stiffness, and improving musculoskeletal health.

In terms of psychological symptoms, the intervention resulted in a significant reduction in worrying, nervousness, irritability, sadness, and anxiety. Psychological distress is prevalent in HD patients due to treatment-related stress, social limitations, and uncertainty about prognosis. The improvement in emotional symptoms suggests that exercise may have contributed to stress reduction, mood enhancement, and improved overall well-being. Exercise has been widely recognized for its positive effects on mental health, primarily through the release of endorphins and other neurochemicals, which help reduce stress and enhance mood (24). Additionally, physical activity promotes better sleep quality, which can further improve emotional stability and overall well-being (48). Studies have also shown that exercise reduces inflammation and oxidative stress, both of which are linked to increased symptoms of anxiety and depression in patients with chronic illnesses (49, 50). For HD patients who engage in exercise at the gym or outdoors, physical activity not only serves as a stress-relief outlet but also fosters social interaction and enhances self-efficacy, both of which have been shown to reduce psychological distress (51). These findings underscore the therapeutic potential of structured exercise programs as an effective non-pharmacological intervention to enhance mental well-being in dialysis patients.

While the present study demonstrated a statistically significant reduction in overall symptom burden following a structured exercise program, it is important to note that not all literature reports consistent improvements across all symptom domains. For instance, some studies have shown that exercise interventions may have limited or no effect on symptoms such as pruritus, sleep disturbances, or certain types of chronic pain (21, 23). These discrepancies may be attributed to differences in exercise modality and duration, intervention intensity, sample size, symptom assessment tools, patient adherence, or even cultural and motivational factors influencing engagement and symptom reporting. Our findings, therefore, support the growing but nuanced evidence that exercise may benefit a broad, though not universal, range of symptoms in HD populations.

The study has several limitations. One key limitation is the reliance on self-reported adherence through weekly phone calls, which may be subject to recall bias, over-reporting or social desirability bias, potentially affecting the accuracy of the data. This could have led participants to overestimate their compliance with the exercise regimen, potentially exaggerating the observed improvements in symptom severity. Additionally, although participants were provided with an exercise guide, relying on unsupervised home exercise may be subject to incorrect performance or insufficient intensity, potentially affecting the accuracy of the intervention’s effectiveness. Additionally, allowing participants to choose when to exercise (during or after dialysis) may have influenced their symptom response and ability to perform or complete the required exercises. Factors such as post-dialysis fatigue, dizziness, and varying motivation levels could differ depending on the timing of the exercise, potentially affecting participants’ performance and adherence. While a reduction in symptom severity was observed before and after the intervention, there was no significant improvement in symptom prevalence. This finding aligns with previous evidence suggesting that the benefits of exercise, particularly in reducing depressive symptoms, are more pronounced with intervention periods extending beyond 4 months (23). Therefore, a longer intervention duration may be necessary to achieve sustained improvements in both symptom severity and prevalence over time. Another limitation is that certain biochemical and physiological parameters that may influence symptom severity, such as serum hemoglobin levels, serum albumin, dialysis adequacy, and inflammatory markers (e.g., C-reactive protein), were not measured. These factors could provide valuable insights into the physiological mechanisms underlying symptom burden and should be considered in future studies. Finally, cultural norms in Saudi Arabia—particularly those related to gender roles, modesty, and limited public access to exercise facilities—may have influenced participants’ willingness and ability to engage in physical activity, potentially affecting intervention outcomes and limiting the generalizability of the findings to other cultural contexts.

### Clinical implications

The findings from this study emphasize the importance of individualized symptom management strategies in HD patients. Given the significant associations between age, gender, dialysis duration, and comorbidities with symptom severity, tailored interventions that address both physiological and psychological needs are essential. The demonstrated benefits of exercise suggest that structured physical activity programs should be integrated into routine care for dialysis patients to help alleviate fatigue, pain, restless legs, and psychological distress.

HD centers may implement structured intradialytic exercise programs, such as low-intensity cycling or resistance band use during the first hour of dialysis, supervised by trained staff. Alternatively, home-based protocols supported by patient education and regular follow-up can offer a low-cost, feasible approach adaptable to diverse clinical and cultural settings.

Furthermore, given that certain symptoms did not improve post-intervention, additional strategies such as nutritional support, pharmacological interventions, and psychological counseling may be necessary for comprehensive symptom management.

Future studies should consider incorporating objective measures such as actigraphy, VO₂ max, or wearable activity trackers to more accurately assess exercise adherence and physiological responses, reducing the bias associated with self-reported outcomes.

## Conclusion

This study highlights the significant impact of demographic and clinical factors on symptom severity in HD patients, as well as the beneficial effects of exercise on both physical and psychological symptoms. While symptoms such as fatigue, restless legs, and emotional distress showed significant improvement post-intervention, others related to metabolic imbalances and fluid overload remained unaffected. These findings underscore the need for a multifaceted approach to symptom management, incorporating exercise, nutritional support, pharmacotherapy, and psychosocial interventions to optimize patient well-being and quality of life. Additionally, we recommend that policymakers support the inclusion of structured exercise in national dialysis care guidelines, clinicians incorporate tailored exercise counseling into routine patient management, and patients be actively encouraged to engage in safe, regular physical activity as part of their symptom management plan.

## Data Availability

The raw data supporting the conclusions of this article will be made available by the authors, without undue reservation.
